# Prognostic Effect of Underlying Chronic Kidney Disease and Renal Replacement Therapy on the Outcome of Patients after Out-of-Hospital Cardiac Arrest: A Nationwide Observational Study

**DOI:** 10.3390/medicina58030444

**Published:** 2022-03-18

**Authors:** Won Yang, Jae-Guk Kim, Gu-Hyun Kang, Yong-Soo Jang, Wonhee Kim, Hyun-Young Choi, Yoonje Lee

**Affiliations:** Department of Emergency Medicine, Kangnam Sacred Heart Hospital, College of Medicine, Hallym University, Seoul 07441, Korea; circle1423@naver.com (W.Y.); drkang9@gmail.com (G.-H.K.); amicoys@gmail.com (Y.-S.J.); wonsee02@gmail.com (W.K.); chy6049@naver.com (H.-Y.C.); yong0831@naver.com (Y.L.)

**Keywords:** out-of-hospital cardiac arrest, chronic kidney insufficiency, prognosis, observational study, emergency department

## Abstract

*Background and Objectives:* This study assessed the prognostic value of underlying chronic kidney disease (CKD) and renal replacement therapy (RRT) on the clinical outcomes from out-of-hospital cardiac arrest (OHCA). *Materials and Methods:* This retrospective study was conducted utilizing the population-based OHCA data of South Korea between 2008 and 2018. Adult (>18 years) OHCA patients with a medical cause of cardiac arrest were included and classified into three categories based on the underlying CKD and RRT: (1) non-CKD group; (2) CKD without RRT group; and (3) CKD with RRT group. A total of 13,682 eligible patients were included (non-CKD, 9863; CKD without RRT, 1778; CKD with RRT, 2041). From the three comparison subgroups, data with propensity score matching were extracted. The influence of CKD and RRT on patient outcomes was assessed using propensity score matching and multivariate logistic regression analyses. The primary outcome was survival at hospital discharge and the secondary outcome was a good neurological outcome at hospital discharge. *Results:* The two CKD groups (CKD without RRT and CKD with RRT) showed no significant difference in survival at hospital discharge compared with the non-CKD group (CKD without RRT vs. non-CKD, *p* > 0.05; CKD with RRT vs. non-CKD, *p* > 0.05). The non-CKD group had a higher chance of having good neurological outcomes than the CKD groups (non-CKD vs. CKD without RRT, *p* < 0.05; non-CKD vs. CKD with RRT, *p* < 0.05) whereas there was no significant difference between the two CKD groups (CKD without RRT vs. CKD with RRT, *p* > 0.05). *Conclusions:* Compared with patients without CKD, the underlying cause of CKD—regardless of RRT—may be linked to poor neurological outcomes. Underlying CKD and RRT had no effect on the survival at hospital discharge.

## 1. Introduction

Cardiac arrest is responsible for almost a quarter of the mortality in individuals receiving renal replacement therapy (RRT) [1,2,3]. Patients with chronic kidney disease (CKD) have a higher rate of arterial wall thickening and stiffening than the normal population, which results in progressive coronary calcification [1,4,5,6]. Additionally, most patients with CKD suffer from malignant arterial hypertension due to inadequate volume control, which is a direct cause of left ventricular hypertrophy (LVH) [7]. LVH is associated with an increased risk of sudden cardiac arrest (SCA), affecting more than 70% of patients receiving RRT [6,8,9,10,11,12,13].

Although overall mortality rates in patients receiving RRT have steadily decreased over the last decade, the rate of sudden cardiac death (SCD) has remained unchanged [14]. Notably, the risk of SCA is increased in CKD patients receiving RRT compared with that in the general population or CKD patients not receiving RRT. However, there has been insufficient research on patients who have suffered out-of-hospital cardiac arrest (OHCA) and are undergoing RRT [15].

As most patients with OHCA have limited information about their renal function before the cardiac arrest, only a few studies have evaluated the prognostic value of underlying CKD and the requirement for RRT in OHCA patients, but these included a small number of patients with CKD [2,13,16]. Thus, in clinical practice, it would be beneficial if the outcomes after cardiac arrest could be predicted using knowledge about the underlying CKD and ongoing RRT. Using nationwide OHCA data, our study examined the relationships between underlying CKD and RRT and the prognosis of patients with OHCA, including survival rates and good neurological outcomes.

## 2. Materials and Methods 

### 2.1. Study Design and Settings

This was a retrospective nationwide population-based observational study that used data from the Out-of-Hospital Cardiac Arrest Surveillance (OHCAS) database maintained by the Korean Centers for Disease Control and Prevention (KCDC) in South Korea from January 2008 to December 2018.

OHCAS was performed in 17 provinces throughout South Korea, a country of 50 million people, and included extensive patient data. The study was conducted according to the guidelines of the Declaration of Helsinki and was approved by the Institutional Review Board of the Kangnam Sacred Heart Hospital in 2021 (IRB No. 2021-09-010). Due to the retrospective nature of the study and the analysis of anonymous clinical data, informed consent was waived. The study methodology followed the checklist for observational studies developed by the Strengthening the Reporting of Observational Studies in Epidemiology. In 2021, the KCDC approved the use of the data for this study.

### 2.2. Data Source

The OHCAS database is a population-based OHCA registry and retrospective patient cohort that is assessed by emergency medical services (EMS). The KCDC provided clinical data on OHCA patients for hospital management and discharge outcomes; information on OHCA patients was acquired from EMS records (http://kdca.go.kr/, accessed on 18 March 2021). KCDC medical record reviewers analyzed the medical records of all OHCA patients admitted to emergency rooms and hospitals.

Patient demographic information such as age, sex, and underlying diseases such as hypertension (HTN), diabetes mellitus (DM), and CKD as well as locations of cardiopulmonary resuscitation (CPR), bystander CPR, post-cardiac arrest care, survival, and neurological outcomes were all gathered through the use of a well-designed survey form. The Utstein Style guidelines and the Resuscitation Outcome Consortium Project were used to create the registry form [17,18].

### 2.3. Study Population

Between January 2008 and December 2018, 293,852 OHCA patients were registered on the OHCAS database. This study included adult patients with OHCA (>18 years of age). The OHCA patients excluded from this study included those with no evidence about the underlying CKD in the medical records or the need for RRT as well as OHCA caused by an unidentified or non-medical cause, under the age of 18, dead on arrival (DOA), do not resuscitate (DNR) status, and uncertain data on survival or neurological outcomes due to data loss during a medical assessment or transfer to other hospitals. 

Patients with OHCA were classified into three groups based on the presence of underlying CKD or the requirement for RRT: (1) non-CKD group, or OHCA patients without CKD as an underlying disease; (2) CKD without RRT group, or OHCA patients with CKD as an underlying disease who did not need RRT; and (3) CKD with RRT group, or OHCA patients with CKD as an underlying disease who were receiving RRT.

### 2.4. Variables

The researchers gathered information on demographics (age, sex), location of cardiac arrest (residential vs. non-residential), bystander CPR, bystander automated external defibrillator (AED) use, etiological variables (cardiac vs. non-cardiac), initial cardiac rhythm (shockable vs. non-shockable), prehospital return of spontaneous circulation (ROSC), underlying disease, and post-cardiac arrest care (primary coronary intervention (PCI) and targeted temperature management (TTM)).

The underlying diseases of patients, including CKD, were defined as those diagnosed by a clinician before cardiac arrest and documented in their medical records. The Supplementary Materials provide a definition and a thorough classification of the underlying diseases (Table S1). Patients undergoing RRT had already received chronic hemodialysis before cardiac arrest. Dialysis (hemodialysis or peritoneal dialysis), hemofiltration, and hemodiafiltration are all types of blood filtration that can be performed with or without the use of machines.

Shockable rhythms include ventricular fibrillation and pulseless ventricular tachycardia. Cardiac function failure such as cardiac tamponade, ischemic heart disorders, arrhythmias, or the presumed cardiac cause of unexpected arrest patients was defined as the cause of cardiac arrest. Angioplasty with a balloon and stent placement was part of the PCI procedure. Any surface or intravascular cooling device with a temperature feedback control system was designated as the TTM cooling method. All TTM protocols in South Korea follow the 2010–2015 worldwide recommendations of the American Heart Association (target temperature: 32–36 °C; maintenance time: 12–24 h) [19,20]. The Glasgow–Pittsburgh cerebral performance categories (CPC) scale was used to assess the neurological outcomes. CPC 1–2 denoted good neurological outcomes whereas CPC 3–5 denoted poor neurological outcomes.

### 2.5. Outcome Measures

The primary outcome was survival at hospital discharge and the secondary outcome was a good neurological outcome (CPC 1 and 2) at hospital discharge. 

### 2.6. Statistical Analysis

For the continuous data, the demographic features were reported as medians and an interquartile range whereas for the categorical data, the frequencies and percentages were used. The Kolmogorov–Smirnov test was used to determine the normality of each continuous variable. Pearson’s chi-squared or Fisher’s exact test were used to compare the categorical variables. The Kruskal–Wallis test was used to compare the continuous variables. We evaluated the impact of CKD and RRT on the outcomes using the stepwise backward elimination approach of a multivariate logistic regression analysis. We also used propensity score-matched samples to adjust for the differences in the baseline characteristics between the patient groups for assessing the influence of CKD and RRT on the outcomes. After using the propensity score-matched sample method, three comparison groups were created: non-CKD vs. CKD without RRT, non-CKD vs. CKD with RRT, and CKD without RRT vs. CKD with RRT.

To minimize the influence of potential confounders and selection bias, propensity score matching was used to compensate for the differences in the baseline patient characteristics between the two groups of patients. To choose the participants in both groups, we used 1:1 propensity score matching (control:treatment = 1:1) at a caliper coefficient of 0.2. The included characteristics as covariates were sex, age, location of cardiac arrest, witnessed cardiac arrest, bystander CPR, bystander automated external defibrillator use, cause of OHCA, initial cardiac rhythm, prehospital ROSC, diabetes mellitus, hypertension, heart disease, respiratory disease, stroke primary coronary intervention, and targeted temperature management. The standardized difference in the means was used in assessing the improvement of the covariate balance after propensity score matching. The initial unmatched and matched samples were assessed by calculating the standardized differences. A standardized difference of less than the absolute value of 0.1 was taken to indicate a negligible difference in the mean or the prevalence of a covariate between the compared groups. A multivariate logistic regression model was used to generate the propensity scores. Nearest neighbor matching was employed as the matching approach. The estimated propensity score difference between the control and treatment groups has the smallest absolute value in this approach of matching. To examine the predictive influence of CKD or RRT on the outcomes of the three propensity score-matched subgroups, we used a multivariate logistic regression analysis.

Any variables having *p* < 0.05 in the univariate analyses were included in the multivariate regression analysis. Adjusted odds ratios and 95% confidence intervals were calculated using multivariate logistic regression models. In the multivariate logistic regression model, stepwise backward elimination was applied. The R package (version 4.1.1, The R foundation for Statistical Computing) and the SPSS version 26.0 program (IBM, Armonk, NY, USA) were used to conduct all the statistical analyses. The statistical significance was set at *p* < 0.05.

## 3. Results

### 3.1. Participant Characteristics

**Figure 1:**
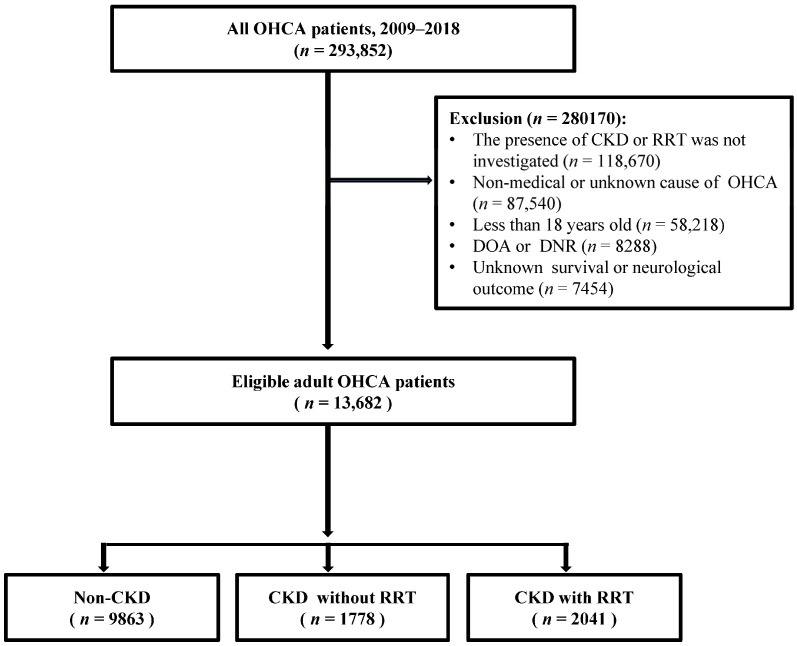
Flow chart of patient inclusions and exclusions in this study. OHCA, out-of-hospital cardiac arrest; CKD, chronic kidney disease; RRT, renal replacement therapy; DOA, dead on arrival; DNR, do not resuscitate.

A total of 293,852 OHCA patients were registered during the study period. The 280,170 patients excluded from this study were patients with no investigation of the underlying CKD or RRT (*n* = 118,670), non-medical or unknown cause of OHCA (*n* = 87,540), age less than 18 years (*n* = 58,218), DOA or DNR (*n* = 8288), and unknown survival or neurologic outcomes (*n* = 7454). Finally, 13,682 patients were found to be eligible (non-CKD: 9863; CKD without RRT: 1778; CKD with RRT: 2041) (Figure 1).

Table 1 outlines the clinical features of all enrolled patients, of whom 2051 (15.0%) had survival at discharge and 1894 (13.8%) were discharged with a good neurological outcome (CPC score of 1 or 2). 

### 3.2. Outcomes Analysis

The non-CKD group had a higher rate of survival at hospital discharge (non-CKD, *n* = 1825 (18.5%) vs. CKD without RRT, *n* = 91 (5.1%) vs. CKD with RRT, *n* = 135 (6.6%); *p* < 0.001) and had better neurological outcomes (non-CKD, *n* = 1711 (17.3%) vs. CKD without RRT, *n* = 73 (4.1%) vs. CKD with RRT, *n* = 110 (5.4%); *p* < 0.001).

### 3.3. Multivariate Logistic Analysis of Survival at Hospital Discharge and Good Neurological Outcomes in the Three Patient Groups

In survival at hospital discharge, the non-CKD group had no better chance than both CKD groups (CKD without RRT and CKD with RRT) (*p* > 0.05). There were no significant differences between the CKD without RRT and CKD with RRT groups in this variable (adjusted OR (95% CI), 0.96 (0.66–1.40); *p* = 0.84) (Table 2). In the good neurological outcome, both CKD groups (CKD without RRT and CKD with RRT) had a lower chance than the non-CKD group (*p* < 0.05). However, there were no significant differences between the CKD without RRT and CKD with RRT groups in this variable (adjusted OR (95% CI), 1.06 (0.71–1.59); *p* = 0.74) (Table 2).

The presence of underlying CKD was associated with a reduced likelihood of good neurologic outcomes, but it had no effect on survival at hospital discharge. Furthermore, RRT was not associated with a higher chance of survival or good neurological outcomes in OHCA patients with CKD.

### 3.4. Subgroup Analysis for Survival at Hospital Discharge and Good Neurological Outcomes According to the Existence of Diabetes Mellitus Using a Multivariate Logistic Analysis

In patients with DM, the presence of CKD and RRT was not associated with survival at hospital discharge and neurological prognosis (*p* > 0.05) (Table 3). In the patients without DM, the non-CKD group was associated with a good neurological outcome compared with the CKD groups (CKD without RRT and CKD with RRT) (*p* < 0.05). There was no significant difference between CKD without RRT and CKD with RRT (adjusted OR (95% CI), 0.91 (0.46–1.80); *p* = 0.80). In contrast, the presence of CKD and RRT was not associated with survival at hospital discharge (*p* > 0.05) (Table 3).

### 3.5. Propensity Score-Matched Analysis of Survival at Hospital Discharge and Good Neurological Outcomes in the Three Patient Groups

Data from the three comparison groupings were matched on propensity scores. The comparison was of non-CKD vs. CKD without RRT (each *n* = 1401), non-CKD vs. CKD with RRT (each *n* = 1541), and CKD without RRT vs. CKD with RRT (each *n* = 1444). (Tables S2–S4). The change of absolute standardized difference in the means and the dot plot of the absolute standardized mean difference illustrated an improvement in the covariate balance after propensity score matching (Figures S1–S3).

### 3.6. Outcome Analysis

The non-CKD patients had no better chance of survival at hospital discharge than the CKD without RRT (non-CKD, *n* = 80 (5.7%) vs. CKD without RRT, *n* = 77 (5.5%); *p* = 0.87) and CKD with RRT (non-CKD, *n* = 108 (7.0%) vs. CKD with RRT, *n* = 102 (6.6%); *p* = 0.72) groups (Tables S2–S4). The non-CKD group had a better likelihood of having good neurological outcomes than the CKD group without RRT (non-CKD, *n* = 71 (5.1%) vs. CKD without RRT, *n* = 46 (3.3%); *p* = 0.023) and CKD with RRT group (non-CKD, *n* = 101 (6.6%) vs. CKD with RRT, *n* = 68 (4.4%); *p* = 0.011) (Tables S2–S4).

### 3.7. Multivariate Logistic Analysis of Survival at Hospital Discharge and Good Neurological Outcomes in the Three Patient Groups after Propensity Score Matching

In survival at hospital discharge, both CKD groups had no better chance than the non-CKD group (CKD without RRT (AOR (95% CI), 0.91 (0.58–1.41); *p* = 0.67) and CKD with RRT (AOR (95% CI), 0.87 (0.61–1.24); *p* = 0.46). There were no significant differences between the CKD without RRT and CKD with RRT groups in this variable (AOR (95% CI), 1.07 (0.73–1.59); *p* = 0.70) (Table 4). In good neurological outcomes, both CKD groups had a lower chance than the non-CKD group (CKD without RRT (AOR [95% CI], 0.45 [0.27–0.75]; *p* < 0.01)) and CKD with RRT (AOR (95% CI), 0.53 (0.36–0.79); *p* < 0.01). However, there were no significant differences between the CKD without RRT and CKD with RRT groups in this variable (AOR (95% CI), 1.16 (0.74–1.81); *p* = 0.50) (Table 4). 

The presence of underlying CKD was associated with a reduced likelihood of good neurologic outcomes, but it had no effect on survival at hospital discharge. Furthermore, among patients with CKD, RRT was not associated with a better chance of survival at hospital discharge or having good neurological outcomes.

## 4. Discussion

This study showed that underlying CKD was associated with poor neurological outcomes in OHCA patients regardless of whether they were undergoing RRT. However, both underlying CKD and RRT showed no prognostic impact on the survival at hospital discharge. 

CKD patients make up a significant proportion of cardiac arrest patients and, irrespective of whether they are undergoing RRT, they are known to have a worse prognosis than non-CKD patients [13]. According to Sherif et al., the progression of CKD is related to a decrease in cardiac repolarization, as evidenced by two-thirds of CKD patients showing a QT prolongation on an electrocardiogram. The prolongation was affected by electrolyte imbalances, drug ingestion, or underlying structural heart disease [21]. Furthermore, this pro-arrhythmogenic adverse effect has been linked to sudden potassium and calcium shifts during RRT [4,6]. As a result, CKD and RRT can cause electrical abnormalities in the heart, frequently leading to fatal arrhythmia. In addition, increased blood pressure and volume load in CKD patients may also contribute to a background of mechanical and electrical imbalances in myocytes, thereby increasing the likelihood of ventricular tachycardia [22,23,24]. Therefore, CKD patients may have a higher probability of cardiac arrest than non-CKD patients because of the high risk of fatal arrhythmia. Previous studies have focused on the risk of cardiac arrest in patients with CKD. However, few studies have evaluated the prognostic impact of underlying CKD or RRT on the outcome of a patient when cardiac arrest occurred; the number of patients included in these studies was small [2,25].

In this study, underlying CKD was associated only with poor neurological outcomes and not with survival. The decreased kidney dysfunction in CKD can affect the permeability of the blood–brain barrier [26]; thus, the protective effect of the blood–brain barrier against several noxious materials after cardiac arrest may be reduced in CKD patients. The level of brain edema caused by an ischemia-reperfusion injury may vary depending on the renal function. As a result, underlying CKD may exacerbate the hypoxic-ischemic insult to the brain after cardiac arrest [26,27]. These clinical traits of CKD may contribute to deteriorated clinical outcomes in OHCA patients. In our research, the CKD group also had higher underlying disease rates (DM, HTN, heart disease, and stroke) than the non-CKD group. Furthermore, we discovered that the CKD group had a lower proportion of features such as a shockable rhythm and prehospital ROSC that were associated with a better prognosis than the non-CKD group [28,29,30]. These unfavorable prehospital factors can also contribute to the poor neurological outcome of CKD patients. In contrast to this study, Weidner et al. showed that CKD with and without RRT is an independent predictor of in-hospital death and long-term all-cause mortality after two years [13]. However, even though Weidner et al. evaluated the long-term survival of patients, their study also included patients presenting with ventricular tachycardia and in-hospital cardiac arrest (IHCA) in addition to OHCA patients; the IHCA patients generally had better outcomes than the OHCA patients [31]. Therefore, the different results between the two studies were assumed to be caused by the disparity in the included population. 

Kim et al. reported that end-stage renal disease, which requires RRT, was a strong risk factor for a poor neurological outcome when providing TTM to initial survivors of OHCA [32]. However, in this study, RRT showed no prognostic effect on the outcome of CKD patients. This difference may be caused by the lower rates of TTM. Given the neuroprotective effect of TTM, a lower rate of TTM made no difference in the outcome of the CKD without RRT and CKD with RRT groups. Another possible explanation could be the low rates of PCI in the CKD group. In adults with OHCA, renal impairment is associated with an increased risk of cardiovascular diseases [33,34,35] and cardiac causes, predominantly acute myocardial infarction and ventricular arrhythmia, are the most common etiologies of cardiac arrest [36,37,38] in patients with renal impairment. The non-significant difference in the neurological outcomes among CKD patients may have been caused by the lower rate of reperfusion treatment.

The specific causes of the low TTM and PCI ratios in this study were unknown because the related data were not investigated. Nevertheless, there were several possible explanations for the estimate. TTM and PCI devices as well as medical staff are not available in all hospitals and university hospitals with TTM and PCI devices and medical staff have a limited number of employees and equipment. Furthermore, TTM and PCI are two of the most expensive medical procedures in South Korea. Therefore, given the prognostic effects of TTM and PCI, other studies with similar populations but high rates of TTM or PCI could show different results compared with this study. 

Males were shown to have higher levels of OHCA than females in all three groups in this study. According to previous research that assessed the influence of sex on cardiac arrest outcomes [39,40,41], men are overrepresented in the cardiac arrest population, accounting for roughly 60% of all occurrences. These differences in OHCA incidence between men and women may be due to differences in the underlying etiology of the disease. In a recent autopsy investigation, women were found to be more likely than men to have non-ischemic causes of autopsy-defined sudden arrhythmic death [42].

This study has certain limitations. First, because this was a retrospective observational study, we could not completely rule out the possibility of reporting and selection bias even though we used propensity score matching to decrease these biases. As a result, these findings should be interpreted with caution. Second, the CKD and RRT information was based on medical records, but the grades of CKD, duration of RRT, and type of RRT (peritoneal dialysis or hemodialysis) were not available, which could have affected the patient outcomes. Third, the long-term outcomes after discharge from the hospital were not evaluated. As a result, the rates of survival and favorable neurological outcomes in this study may differ from those in other studies that evaluated the long-term outcomes of OHCA patients. Fourth, because related information was not available in the registry, we did not assess time intervals such as the response time and CPR duration. This component can be a major determinant of survival and neurological prognosis, both of which have an impact on the patient outcomes. As a result, well-designed investigations with other variables such as response time and CPR duration should be conducted to corroborate the findings of this study. Finally, because the medical systems and medical resources—especially EMS and resuscitation techniques—differ among nations, these conclusions cannot be applied to other nations and similar research findings in other countries may differ from those of this study. However, in the current study, we used a standardized Utstein data collection template and accounted for the hospital differences in all analytical models, thus avoiding a few of these difficulties.

## 5. Conclusions

The presence of underlying CKD may be associated with worse neurological outcomes in OHCA patients, independent of RRT, when compared with patients without CKD. The presence of CKD and RRT had no effect on survival at hospital discharge. Further research is needed to corroborate these findings by assessing the long-term effects and integrating more detailed in-hospital patient data.

## Figures and Tables

**Table 1:** Demographic characteristics of the study population according to chronic renal disease and renal replacement therapy.

Variables	Total	Non-CKD	CKD without RRT	CKD with RRT	*p*-Value
	(*n* = 13682)	(*n* = 9863)	(*n* = 1778)	(*n* = 2041)	
Sex, *n* (%) Male	9377 (68.5%)	7037 (71.3%)	1077 (60.6%)	1263 (61.9%)	<0.01
Age, years (IQR)	64 (52–76)	61 (49–75)	75 (66–81)	66 (57–74)	<0.01
Location of CA					<0.01
Residential CA	7414 (54.2%)	5152 (52.2%)	1106 (62.2%)	1156 (56.6%)	
Non-residential CA	6268 (45.8%)	4711 (47.8%)	672 (37.8%)	885 (43.4%)	
Witnessed CA	8426 (61.6%)	6167 (62.5%)	1033 (58.1%)	1226 (60.1%)	<0.01
Bystander CPR	3684 (26.9%)	2936 (29.8%)	383 (21.5%)	365 (17.9%)	<0.01
Bystander AED use	39 (0.3%)	33 (0.3%)	1 (0.1%)	5 (0.2%)	<0.01
Cause of CA					<0.01
Cardiac origin	12723 (93.0%)	9128 (92.5%)	1702 (95.7%)	1893 (92.7%)	
Non-cardiac origin	959 (7.0%)	735 (7.5%)	76 (4.3%)	148 (7.3%)	
Initial cardiac rhythm Shockable					<0.01
	2814 (20.6%)	2470 (25.1%)	148 (8.3%)	196 (9.6%)	
Non-shockable	10,868 (79.4%)	7393 (74.9%)	1630 (91.7%)	1845 (90.4%)	
Prehospital ROSC	2075 (15.2%)	1817 (18.4%)	105 (5.9%)	153 (7.5%)	<0.01
Underlying disease					
DM	3158 (23.1%)	878 (8.9%)	1050 (59.1%)	1230 (60.3%)	<0.01
HTN	4411 (32.2%)	2028 (20.6%)	1115 (62.7%)	1268 (62.1%)	<0.01
Dyslipidemia	320 (2.3%)	195 (2.0%)	83 (4.7%)	42 (2.1%)	<0.01
Heart disease	1907 (13.9%)	603 (6.1%)	672 (37.8%)	632 (31.0%)	<0.01
Respiratory disease	534 (3.9%)	285 (2.9%)	149 (8.4%)	100 (4.9%)	<0.01
Stroke	658 (4.8%)	256 (2.6%)	218 (12.3%)	184 (9.0%)	<0.01
Post-CA care					
PCI	1097 (8.0%)	979 (9.9%)	55 (3.1%)	63 (3.1%)	<0.01
TTM	1017 (7.4%)	824 (8.4%)	69 (3.9%)	124 (6.1%)	<0.01
Outcomes at hospital discharge					
Survival	2051 (15.0%)	1825 (18.5%)	91 (5.1%)	135 (6.6%)	<0.01
Good neurological outcome	1894 (13.8%)	1711 (17.3%)	73 (4.1%)	110 (5.4%)	<0.01

CKD, chronic kidney disease; RRT, renal replacement therapy; IQR, interquartile range; CA, cardiac arrest; CPR, cardiopulmonary resuscitation; AED, automated external defibrillator; DM, diabetes mellitus; HTN, hypertension; PCI, primary coronary intervention; TTM, targeted temperature management; ROSC, return of spontaneous circulation.

**Table 2:** Multivariate logistic regression analysis by chronic kidney disease and renal replacement therapy on outcomes.

Outcome	Groups	Reference = Non-CKD				Reference = CKD without RRT			
		AOR	95% CI		*p*-Value	AOR	95% CI		*p*-Value
Survival at hospital discharge *	Non-CKD	1.00				1.01	0.71	1.44	0.93
	CKD without RRT	0.98	0.69	1.40	0.93	1.00			
	CKD with RRT	0.94	0.69	1.28	0.73	0.96	0.66	1.40	0.84
Good neurological outcome *	Non-CKD	1.00				1.79	1.24	2.58	<0.01
	CKD without RRT	0.55	0.38	0.80	<0.01	1.00			
	CKD with RRT	0.59	0.43	0.81	<0.01	1.06	0.71	1.59	0.74

CKD, chronic kidney disease; RRT, renal replacement therapy; AOR, adjusted odds ratio; CI, confidence interval. * Adjusted odds ratio for sex, age, location of cardiac arrest, witnessed cardiac arrest, bystander CPR, bystander automated external defibrillator use, cause of OHCA, initial cardiac rhythm, prehospital ROSC, diabetes mellitus, hypertension, heart disease, respiratory disease, stroke primary coronary intervention, and targeted temperature management.

**Table 3:** Multivariate logistic regression analysis by chronic kidney disease and renal replacement therapy on outcomes according to the presence of diabetes mellitus.

Outcome	Groups	Reference = Non-CKD				Reference = CKD without RRT			
		AOR	95% CI		*p*-Value	AOR	95% CI		*p*-Value
Patients with DM									
Survival at hospital discharge *	Non-CKD	1.00				0.88	0.55	1.42	0.61
	CKD without RRT	1.12	0.70	1.82	0.61	1.00			
	CKD with RRT	1.03	0.67	1.58	0.89	0.91	0.59	1.40	0.67
Good neurological outcome *	Non-CKD	1.00				1.57	0.95	2.59	0.07
	CKD without RRT	0.63	0.38	1.04	0.07	1.00			
	CKD with RRT	0.67	0.43	1.04	0.07	1.06	0.66	1.71	0.78
Patients without DM									
Survival at hospital discharge *	Non-CKD	1.00				1.47	0.84	2.58	0.17
	CKD without RRT	0.67	0.38	1.18	0.17	1.00			
	CKD with RRT	0.68	0.42	1.09	0.11	1.01	0.51	2.00	0.96
Good neurological outcome *	Non-CKD					2.17	1.26	3.73	<0.01
	CKD without RRT	0.46	0.26	0.78	<0.01	1.00			
	CKD with RRT	0.42	0.25	0.68	<0.01	0.91	0.46	1.80	0.80

CKD, chronic kidney disease; DM, diabetes mellitus; RRT, renal replacement therapy; AOR, adjusted odds ratio; CI, confidence interval. * Adjusted odds ratio for sex, age, location of cardiac arrest, witnessed cardiac arrest, bystander CPR, bystander automated external defibrillator use, cause of OHCA, initial cardiac rhythm, prehospital ROSC, hypertension, heart disease, respiratory disease, stroke primary coronary intervention, and targeted temperature management.

**Table 4:** Multivariate logistic analysis for the propensity score-matched patients between three groups for outcomes.

Outcomes	Groups	Non-CKD (Ref.) vs. CKD without RRT (Test)			Non-CKD (Ref.) vs. CKD with RRT (Test)			CKD without RRT (Ref.) vs. CKD with RRT (Test)		
		AOR	95% CI	*p*-Value	AOR	95% CI	*p*-Value	AOR	95% CI	*p*-Value
Survival at hospital discharge *	Ref.	1.00			1.00			1.00		
	Test	0.91	0.58–1.41	0.67	0.87	0.61–1.24	0.46	1.07	0.73–1.59	0.70
Good neurological outcome *	Ref.	1.00			1.00			1.00		
	Test	0.45	0.27–0.75	<0.01	0.53	0.36–0.79	<0.01	1.16	0.74–1.81	0.50

CKD, chronic kidney disease; RRT, renal replacement therapy; AOR, adjusted odds ratio; CI, confidence interval. * Adjusted odds ratio for sex, age, location of cardiac arrest, witnessed cardiac arrest, bystander CPR, bystander automated external defibrillator use, cause of OHCA, initial cardiac rhythm, prehospital ROSC, diabetes mellitus, hypertension, heart disease, respiratory disease, stroke, primary coronary intervention, and targeted temperature management.

## Data Availability

The authors of this study used the data from the Out-of-Hospital Cardiac Arrest Surveillance, 2012, Korea Disease Control and Prevention Agency.

## Supplementary Materials

The following supporting information can be downloaded at: https://www.mdpi.com/article/10.3390/medicina58030444/s1. Table S1: Definition and detailed classification of underlying disease; Table S2: Demographic characteristics of the study population of the two groups (non-CKD and CKD without RRT) after propensity score matching; Table S3: Demographic characteristics of the study population of the two groups (non-CKD and CKD with RRT) after propensity score matching; Table S4: Demographic characteristics of the study population of the two groups (CKD without RRT and CKD with RRT) after propensity score matching; Figure S1: Change of absolute standardized difference in means (A) and dot plot of absolute standardized mean difference (B) in patients with non-CKD and CKD without RRT before and after propensity score matching; Figure S2: Change of absolute standardized difference in means (A) and dot plot of absolute standardized mean difference (B) in patients with non-CKD and CKD with RRT before and after propensity score matching; Figure S3: Change of absolute standardized difference in means (A) and dot plot of absolute standardized mean difference (B) in patients with CKD without RRT and CKD with RRT before and after propensity score matching.
