# Supplementary material for: Prognostic Effect of Underlying Chronic Kidney Disease and Renal Replacement Therapy on the Outcome of Patients after Out-of-Hospital Cardiac Arrest: A Nationwide Observational Study
Source: Medicina (Kaunas). 2022 Mar 18;58(3):444. doi: 10.3390/medicina58030444 (PMC8948889; doi:10.3390/medicina58030444)
Supplement: Supplementary file 1 [file medicina-58-00444-s001.zip › medicina-1609215-supplementary.pdf]

Table S1. Definition and detailed classification of underlying disease.

Disease	Definition and detailed classification
Hypertension	The disease was diagnosed by the doctor prior to cardiac arrest and is clearly stated in the medical record
Diabetes mellitus	The disease was diagnosed by the doctor prior to cardiac arrest and is clearly stated in the medical record.
Heart disease	The disease was diagnosed by the doctor prior to cardiac arrest and is clearly stated in the medical record.<br>(Ischemic heart disease, myocardial infarction, angina, valvular heart disease, arrhythmia, congestive heart disease, myocardiopathy, congenital heart disease, previous heart intervention record including stent, primary coronary intervention, coronary artery bypass grafting, and other heart disease)
Chronic kidney disease	The disease was diagnosed by the doctor prior to cardiac arrest and is clearly stated in the medical record.<br>(chronic renal failure, dialysis, kidney transplantation, and other chronic kidney disease)
Chronic respiratory disease	The disease was diagnosed by the doctor prior to cardiac arrest and is clearly stated in the medical record. (asthma, chronic obstructive pulmonary disease, pulmonary tuberculosis, other chronic respiratory disease.)
Stroke	The disease was diagnosed by the doctor prior to cardiac arrest and is clearly stated in the medical record.<br>(cerebral infarction, cerebral haemorrhage, and unclassified stroke)
Dyslipidemia	The disease was diagnosed by the doctor prior to cardiac arrest and is clearly stated in the medical record.<br>(hyperlipidaemia and unclassified dyslipidemia)

Table S2. Demographic characteristics of the study population of two groups (non-CKD and CKD without RRT) after propensity score matching.

Variables	Non-CKD (N=1,401)	CKD without RRT (N=1,401)	*P-value*
**Sex, n (%)**			
Male	845 (60.3%)	844 (60.2%)	1.000
**Age, years [median (IQR)]**	75.0 [64.0-81.0]	75.0 [65.0-81.0]	0.681
**Location of CA**			
Residential CA	862 (61.5%)	865 (61.7%)	0.938
Non-residential CA	539 (38.5%)	536 (38.3%)	
**Witnessed CA**	823 (58.7%)	810 (57.8%)	0.646
**Bystander CPR**	298 (21.3%)	316 (22.6%)	0.438
**Bystander AED use**	1 (0.1%)	1 (0.1%)	1.000
**Cause of cardiac arrest**			
Cardiac origin	1,351 (96.4%)	1,340 (95.6%)	0.333
Non-cardiac origin	50 (3.6%)	61 (4.4%)	
**Initial cardiac rhythm**			
Shockable	123 (8.8%)	132 (9.4%)	0.599
None-shockable	1278 (91.2%)	1269 (90.6%)	
**Prehospital ROSC**	95 (6.8%)	92 (6.6%)	0.880
**Underlying disease**			
DM	689 (49.2%)	710 (50.7%)	0.450
HTN	780 (55.7%)	788 (56.2%)	0.790
Dyslipidemia	52 (3.7%)	56 (4.0%)	0.768
Heart disease	384 (27.4%)	436 (31.1%)	**0.034**
Resp. disease	110 (7.9%)	117 (8.4%)	0.678
Stroke	138 (9.9%)	160 (11.4%)	0.198
**Post-cardiac arrest care**			
PCI	78 (5.6%)	85 (6.1%)	0.628
TTM	52 (3.7%)	56 (4.0%)	0.768
**Outcomes at hospital discharge**			
Survival	80 (5.7%)	77 (5.5%)	0.870
Good neurological outcome	71 (5.1%)	46 (3.3%)	**0.023**

Abbreviations: CKD, chronic kidney disease; RRT, renal replacement therapy; IQR, interquartile range; CA, cardiac arrest; CPR, cardiopulmonary resuscitation; AED, automated external defibrillator; DM, diabetes mellitus; HTN, hypertension; PCI, primary coronary intervention; TTM, targeted temperature management; and ROSC, return of spontaneous circulation.

Table S3. Demographic characteristics of the study population of two groups (non-CKD and CKD with RRT) after propensity score matching.

Variables	Non-CKD (N=1,541)	CKD with RRT (N=1,541)	*P-value*
**Sex, n (%)**			
Male	986 (64.0%)	956 (62.0%)	0.279
**Age, years [median (IQR)]**	69.0 [58.0-78.0]	66.0 [56.0-74.0]	**< 0.001**
**Location of CA**			
Residential CA	863 (56.0%)	871 (56.5%)	0.799
Non-residential CA	678 (44.0%)	670 (43.5%)	
**Witnessed CA**	928 (60.2%)	926 (60.1%)	0.971
**Bystander CPR**	278 (18.0%)	298 (19.3%)	0.380
**Bystander AED use**	2 (0.1%)	5 (0.3%)	0.453
**Cause of cardiac arrest**			
Cardiac origin	1,448 (94.0%)	1,428 (92.7%)	0.171
Non-cardiac origin	93 (6.0%)	113 (7.3%)	
**Initial cardiac rhythm**			
Shockable	152 (9.9%)	163 (10.6%)	0.552
None-shockable	1389 (90.1%)	1378 (89.4%)	
**Prehospital ROSC**	120 (7.8%)	123 (8.0%)	0.894
**Underlying disease**			
DM	750 (48.7%)	740 (48.0%)	0.746
HTN	758 (49.2%)	807 (52.4%)	0.084
Dyslipidemia	41 (2.7%)	32 (2.1%)	0.343
Heart disease	338 (21.9%)	378 (24.5%)	0.096
Resp. disease	76 (4.9%)	76 (4.9%)	1.000
Stroke	111 (7.2%)	132 (8.6%)	0.181
**Post-cardiac arrest care**			
PCI	100 (6.5%)	103 (6.7%)	0.885
TTM	76 (4.9%)	84 (5.5%)	0.570
**Outcomes at hospital discharge**			
Survival	108 (7.0%)	102 (6.6%)	0.721
Good neurological outcome	101 (6.6%)	68 (4.4%)	**0.011**

Abbreviations: CKD, chronic kidney disease; RRT, renal replacement therapy; IQR, interquartile range; CA, cardiac arrest; CPR, cardiopulmonary resuscitation; AED, automated external defibrillator; DM, diabetes mellitus; HTN, hypertension; PCI, primary coronary intervention; TTM, targeted temperature management; and ROSC, return of spontaneous circulation.

Table S4. Demographic characteristics of the study population of two groups (CKD without RRT and CKD with RRT) after propensity score matching.

Variables	CKD without RRT (N=1,444)	CKD with RRT (N=1,444)	*P-value*
**Sex, n (%)**			
Male	882 (61.1%)	890 (61.6%)	0.789
**Age, years [median (IQR)]**	72.0 [63.0-79.0]	70.0 [62.0-76.0]	**< 0.001**
**Location of CA**			
Residential CA	856 (59.3%)	864 (59.8%)	0.791
Non-residential CA	588 (40.7%)	580 (40.2%)	
**Witnessed CA**	853 (59.1%)	852 (59.0%)	1.000
**Bystander CPR**	282 (19.5%)	278 (19.3%)	0.888
**Bystander AED use**	1 (0.1%)	2 (0.1%)	1.000
**Cause of cardiac arrest**			
Cardiac origin	1373 (95.1%)	1377 (95.4%)	0.794
Non-cardiac origin	71 (4.9%)	67 (4.6%)	
**Initial cardiac rhythm**			
Shockable	132 (9.1%)	135 (9.3%)	0.898
None-shockable	1312 (90.9%)	1309 (90.7%)	
**Prehospital ROSC**	92 (6.4%)	91 (6.3%)	1.000
**Underlying disease**			
DM	866 (60.0%)	883 (61.1%)	0.542
HTN	906 (62.7%)	891 (61.7%)	0.591
Dyslipidemia	49 (3.4%)	36 (2.5%)	0.186
Heart disease	501 (34.7%)	492 (34.1%)	0.754
Resp. disease	89 (6.2%)	80 (5.5%)	0.526
Stroke	166 (11.5%)	149 (10.3%)	0.340
**Post-cardiac arrest care**			
PCI	77 (5.3%)	82 (5.7%)	0.744
TTM	64 (4.4%)	61 (4.2%)	0.855
**Outcomes at hospital discharge**			
Survival to hospital discharge	84 (5.8%)	80 (5.5%)	0.809
Good neurological outcome	67 (4.6%)	59 (4.1%)	0.524

Abbreviations: CKD, chronic kidney disease; RRT, renal replacement therapy; IQR, interquartile range; CA, cardiac arrest; CPR, cardiopulmonary resuscitation; AED, automated external defibrillator; DM, diabetes mellitus; HTN, hypertension; PCI, primary coronary intervention; TTM, targeted temperature management; and ROSC, return of spontaneous circulation.

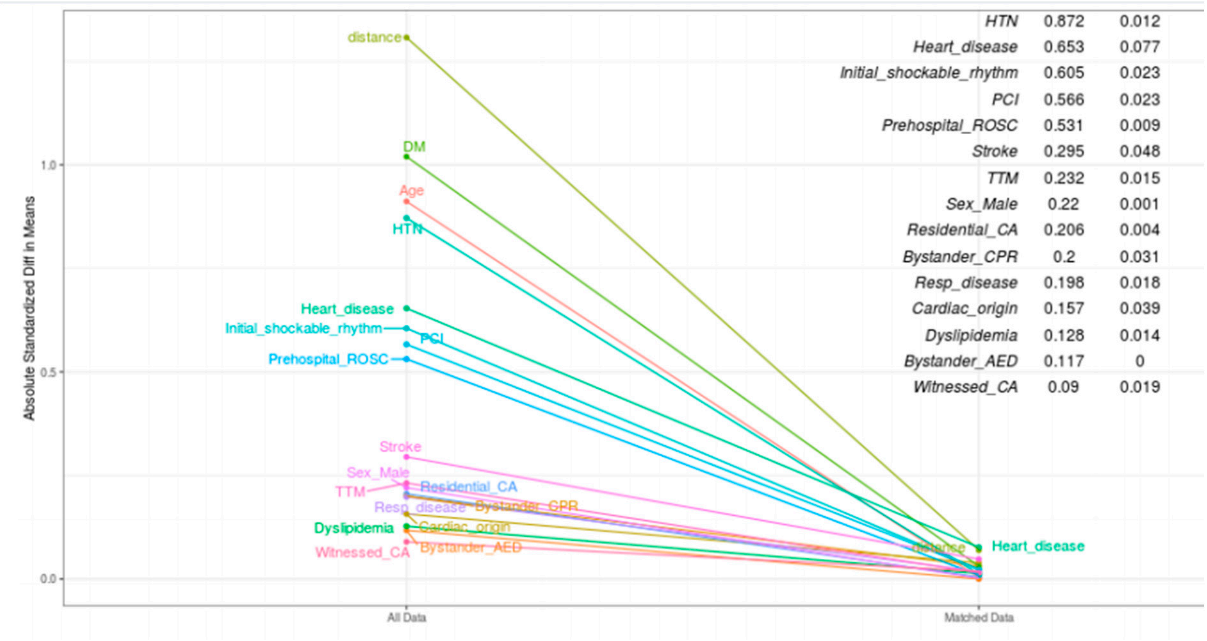

(A)

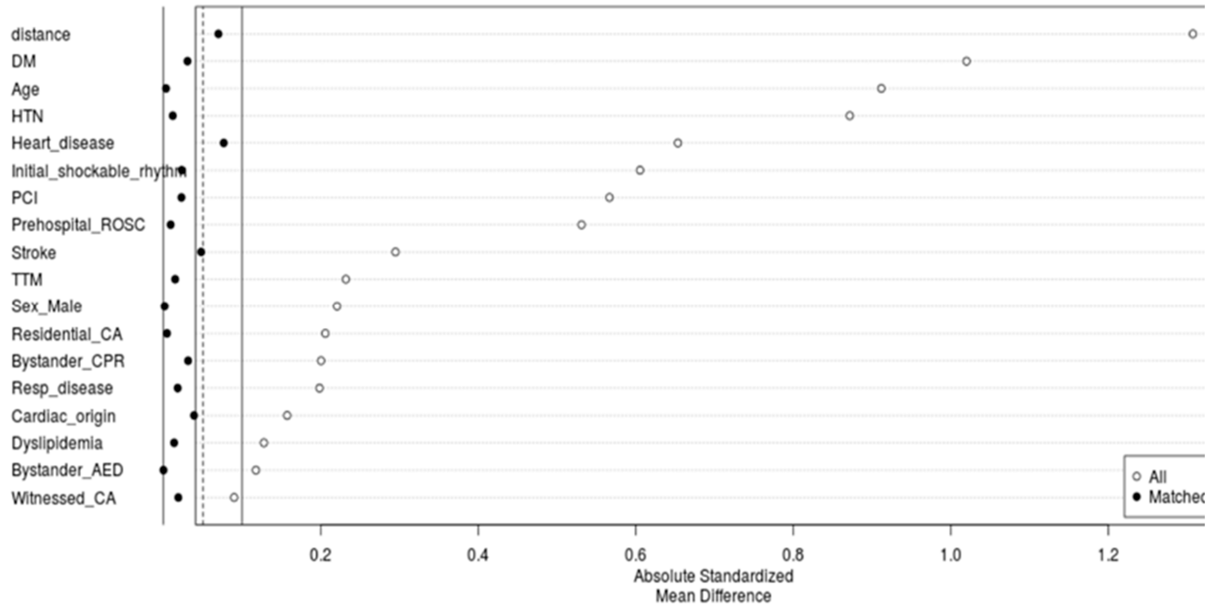

(B)

Figure S1. Change of absolute standardized difference in means (A) and dot plot of absolute standardized mean difference (B) in patients with Non-CKD and CKD without RRT before and after propensity score matching.

Abbreviations: CKD, chronic kidney disease; RRT, renal replacement therapy; CA, cardiac arrest; CPR, cardiopulmonary resuscitation; AED, automated external defibrillator; DM, diabetes mellitus; HTN, hypertension; PCI, primary coronary intervention; TTM, targeted temperature management; and ROSC, return of spontaneous circulation.

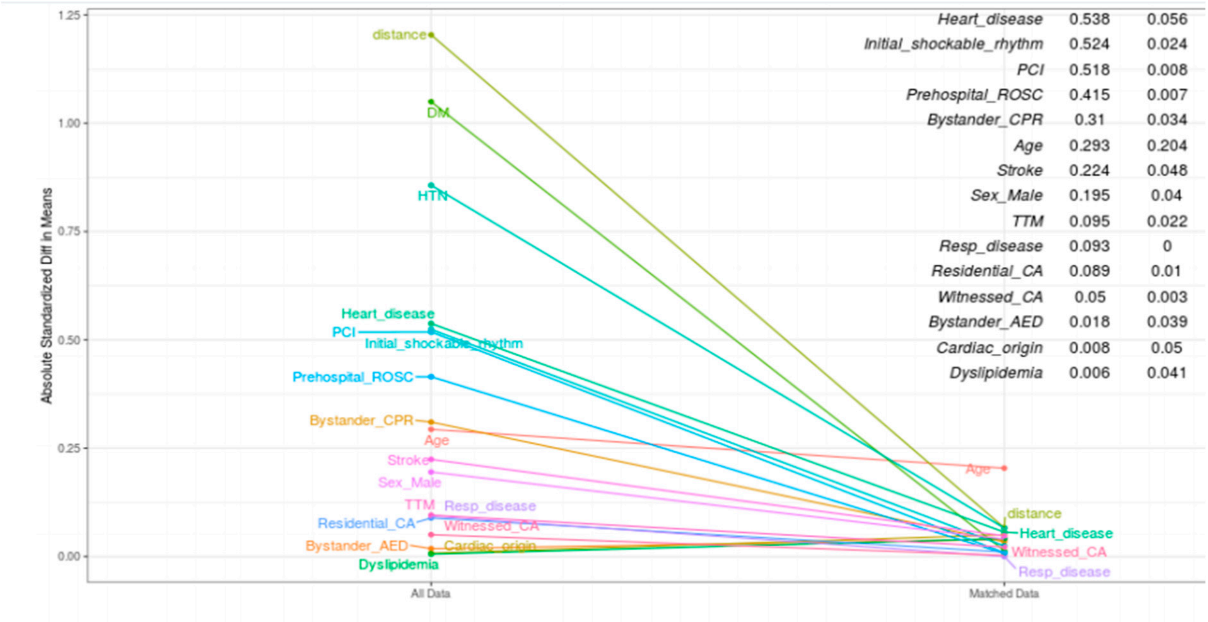

(A)

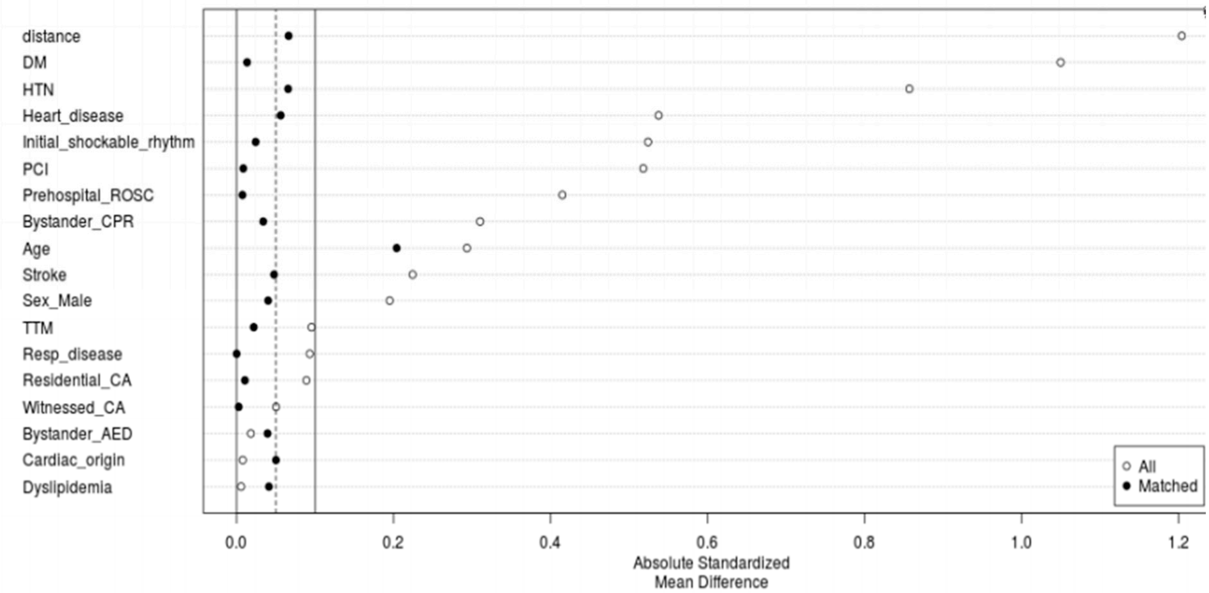

(B)

Figure S2. Change of absolute standardized difference in means (A) and dot plot of absolute standardized mean difference (B) in patients with Non-CKD and CKD with RRT before and after propensity score matching.

Abbreviations: CKD, chronic kidney disease; RRT, renal replacement therapy; CA, cardiac arrest; CPR, cardiopulmonary resuscitation; AED, automated external defibrillator; DM, diabetes mellitus; HTN, hypertension; PCI, primary coronary intervention; TTM, targeted temperature management; and ROSC, return of spontaneous circulation.

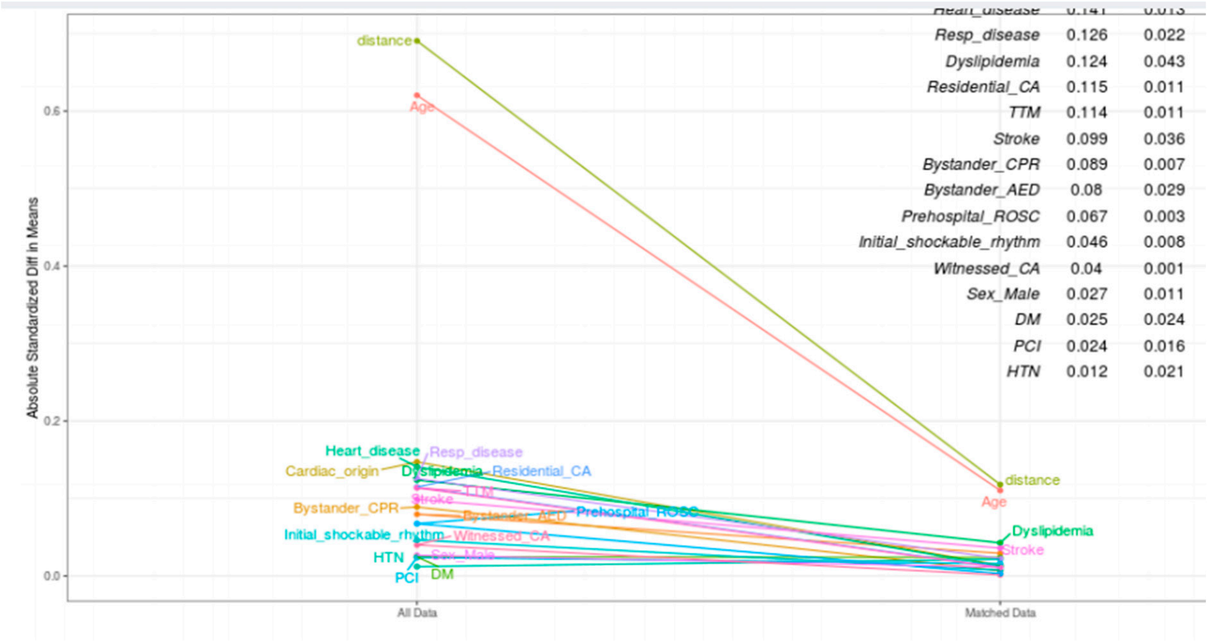

(A)

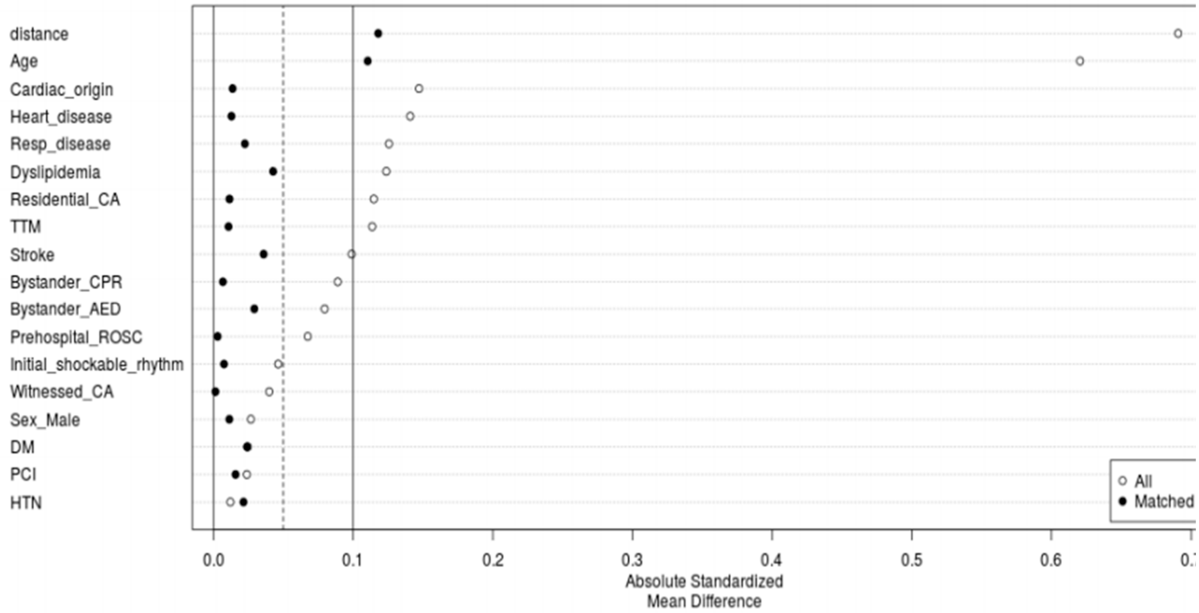

(B)

Figure S3. Change of absolute standardized difference in means (A) and dot plot of absolute standardized mean difference (B) in patients with CKD without RRT and CKD with RRT before and after propensity score matching.

Abbreviations: CKD, chronic kidney disease; RRT, renal replacement therapy; CA, cardiac arrest; CPR, cardiopulmonary resuscitation; AED, automated external defibrillator; DM, diabetes mellitus; HTN, hypertension; PCI, primary coronary intervention; TTM, targeted temperature management; and ROSC, return of spontaneous circulation.
